# Complementary medicine and chronic disease: preliminary results

**DOI:** 10.1186/1824-7288-40-S1-A70

**Published:** 2014-08-11

**Authors:** Gianfranco Trapani, Luisella Zanino, Gianna Gabbanelli, Stefano Gandus, Maurizio Annibalini, Pietro Prandi, Gaetano M Miccichè, Maurizio Calzavara, Domenico Careddu, Sara Griseri, Mariuccia Ventura, Isabella Villa, Illary Sbizzera, GianPaolo Salvioli

**Affiliations:** 1 Pediatra - Società di Medicina Bioterapica - SMB Italia, Roma, Italy; 2 Pediatra di Libera scelta, Italy; 3 Dottore in Scienze Statistiche, Verona, Italy; 4 Professore Emerito Di Pediatria all'Università di Bologna, Bologna, Italy

## Background

The Study Group in Complementary and Alternative Medicines of the Italian Society of Pediatrics (SIP) has an ongoing study to assess how the relationship between families and pediatricians, who also prescribe Complementary and Alternative Medicine (CAM) affect on therapies of children with Chronic Diseases (CD) The study, which began in January 2014, will end in July 2014.

## Materials and methods

Pediatricians administered a questionnaire to families with children affected by CD when they go into ambulatory. The questionnaire assesses the role of CAM requested by the family with regard to the natural history of the disease and the possible Adverse Effect (EA).

They came to our observation (March 2014) 121 Questionnaires. Among these, 109 (90.1%) were found to be suitable for the study. The diseases most frequently identified were: diabetes, inflammatory bowel disease (IBD), cancers, celiac disease, rheumatic diseases, disorders of conduct (DC) such as ADHD, autism, and other DC, cystic fibrosis, and epilepsy.

In addition, 27 families had 36 children (33.0% of cases) affected by other diseases, among them S. Peutz Jeghers, S. Kartegener, primary TBC complex, visually impaired ocular albinism (X gene locus 22), acute lymphoblastic leukemia with removal of colon and rectal anastomosis ileum, Beta sarcoglycanopathy, May Hegglin syndrome (hereditary thrombocytopenia).

## Results

The families who consulted a pediatrician expert in CAM and conventional therapies (CT) have associated the two therapeutic techniques in 80, 7% (88 of 109 cases). 19.3% (21 of 109 cases) decided to leave temporarily or permanently therapies that followed, replacing them with the CAM. Of the 21 families who have abandoned CT, 13 (61.9%) had children with ADHD, autism and other DC, others have declared that have not been proposed therapies, but recommended "watchful waiting" (IBD, autoimmune inflammation of the thyroid, thrombocytopenia).

Of the 109 cases, 31.1% of parents [1] used CAM to enhance the effect of conventional therapy on the disease. 30.2% were not satisfied with the results obtained with the TC on the disease . 37.6% preferred to use CAM during acute illnesses not to take too many drugs .

## Conclusion

Unlike the literature [2,3] No EA was observed for CAM use in 109 cases observed
